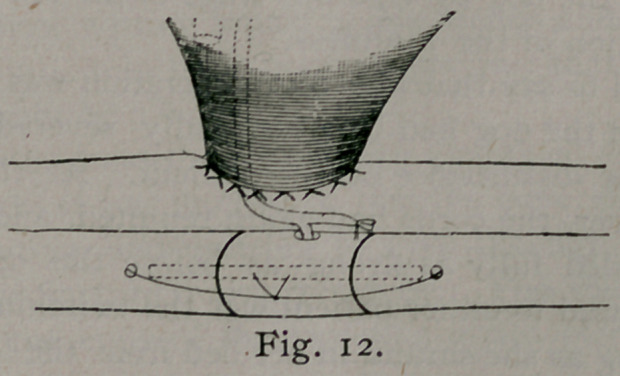# Transplantation of Tissue from Lower Animals to Man

**Published:** 1891-03

**Authors:** A. M. Phelps

**Affiliations:** New York, Professor of Surgery University of Vermont; Professor of Orthopedic Surgery Medical Department University of the City of New York; Professor Orthopedic Surgery New York Post Graduate School and Hospital; Surgeon in Charge of Orthopedic Wards, Visiting Surgeon to Charity Hospital; Consulting Surgeon Mary Fletcher Hospital, etc.


### DANIEL'S
### TEXAS MEDICAL JOURNAL.

A Representative Organ of the Medical Profession, and an Exponent of Rational Medicine; devoted to the Organization, Advancement and Elevation of the Profession in Texas.

PUBLISHED MONTHLY.—SUBSCRIPTION $2.00 A YEAR.

Vol. VI. AUSTIN, MARCH, 1891. No. 9.

### Original Articles.

CONTRIBUTED EXCLUSIVELY TO THIS JOURNAL.

*The Articles in this Department are accepted and published with the understanding that we are not responsible for, nor do we indorse the views and opinions of the writers by so doing.*

---

For Daniel's Texas Medical Journal.

### TRANSPLANTATION OF TISSUE FROM LOWER ANIMALS TO MAN.

### Abstract of the Case of Bone-Transplantation at Charity Hospital, Blackwell's Island, N. Y.

BY A. M. PHELPS, M. D., NEW YORK,

Professor of Surgery University of Vermont; Professor of Orthopedic Surgery Medical Department University of the City of New York; Professor Orthopedic Surgery New York Post Graduate School and Hospital; Surgeon in Charge of Orthopedic Wards, Visiting Surgeon to Charity Hospital; Consulting Surgeon Mary Fletcher Hospital, etc.

THE CASE of transplantation from an animal, recently performed at Charity Hospital, has commanded a wide-spread attention, and all sorts of absurd rumors have been circulated.

The operation is a success in so far as it establishes the principle that it is possible to grow large masses of tissue from an animal to man, and to establish the circulation until the union takes place between opposite species without danger to either. It also demonstrates that a growth of new bone takes place when a section of bone is transplanted and its nutrition maintained by the artery of the animal. This, if continued four or five weeks, would probably unite a fracture. Owing to the inefficient dressings which is apt to occur in early operations, the contact of the transplanted bone could not be continued sufficiently long for bone to unite to bone. But I am confident, after viewing the specimen, and taking all the conditions and surroundings into account, that bony union would have taken place if actual contact had been maintained for a longer period. The stimulation of the graft, however, has excited a reparative process in the fracture, and it now promises fair to unite. The boy walks with the aid of one crutch, or a cane.*

### HISTORY OF THE CASE.

In the month of November, last year, the patient at Charity Hospital was sent to me for operation. Briefly the history of the case is this:

The lad, John Gethins, was suffering from an ununited fracture of the lower third of the leg, the result of an operation to remedy an anterior curve of the tibia, which had existed and had slowly increased from early childhood, until he was compelled to go upon crutches.

Fig. 5 is a photograph of the case before the operation of osteotomy. There was no paralysis of the limb, neither was it atrophied, excepting from non-use. The muscles were perfect in every respect.

This case was referred to my clinic two years ago, by Drs.

---

*A similar operation had been previously made by Dr. Phelps, but it failed; the details of the first case and the illustrations are omitted for want of room. The JOURNAL is indebted to Dr. W. C. Wile, of the New England Medical Journal, for the paper and illustrations. It appears simultaneously in the New England Medical Journal.—ED.

Wey and Flood, of Elmira, and Assemblyman Dr. Bush, of Horsehead.

A few months after the operation of osteotomy, I cut down upon the fracture and wired it, but failed in getting union. After a few months I again operated, removing all cicatricial tissue, carefully stitching the periosteum together, and wired the bone. This failed. A few months later I again cut down upon the fracture, removed all cicatricial tissue, and again freshened the ends of the bone, and engrafted decalcified bone chips, according to Senn's method. This failed; the chips came away from the wound a few weeks after the operation. I then resorted to Thomas' method of hammering, damming, and adjusting an appliance for the boy to walk upon. This he wore for several months, but again we failed to secure union.

Fig. 5.

In the mean time the boy had returned to his home. From time to time I received letters from the lad, beseeching me again to make an effort to restore his limb to usefulness, and avoid an amputation. He returned again to New York, and was referred to one of the largest and best hospitals in the city; and one of the most eminent surgeons of this country operated upon him twice, with a failure each time. The poor boy, discouraged, returned to his home again.

Last fall, in October, I received a most pathetic letter from him, asking if I would again attempt to unite the fracture. A letter was written to him stating that an attempt at bone-transplantation from a dog, of which I had previously told him, would be tried if he desired. In answer to the letter, his parents at once sent him to New York. He was sent to Charity Hospital. The limb, at the time of the operation, November 16, 1890, was in very good condition, excepting the shortening of about four inches, the result of previous operations, which had been performed to unite the fracture.

It seemed a pity to amputate the leg. And surely we had arrived at that stage of the case where, so far as our present knowledge was concerned, amputation was the last and only step to be taken. With all of these facts before us, we felt perfectly justified in attempting aay experiment which would promise to succeed in restoring the limb to usefulness, provided the danger to life was not too great.

The brave lad had submitted to every means known to surgery for the relief of his condition, and, discouraged and heart-broken, had returned to his home. When he was told there was another chance for his limb, his face brightened, and he said, "Doctor, I will take that chance." After consulting with the members of the Medical Board of Charity Hospital, and several eminent surgeons of this city, we decided upon an operation.

It is a well-known surgical fact that an amputation performed in the growing limb below the knee or in the humerus frequently results in what is known as a conical stump. This necessarily leads to re-amputation, and many amputations have been performed from year to year, in the same case, for this abnormality. This was one of the reasons, but not the greatest, why we hesitated to amputate the limb.

A dog two years old was secured and prepared for the operation, carefully cleansed with soap and water, and made aseptic with a solution of bichloride of mercury.

While the patient was being anæsthetised and the ends of the fractured bones freshened, my assistants, Drs. Plimpton and Mooney, prepared the dog, in the following manner: She was etherized, and then enveloped in a thick layer of absorbant cotton to the thickness of several inches, while placed in the natural sitting posture. Over this soft covering of cotton a few turns of a plaster-of-Paris bandage were made, to hold the dressing in place. The dog was not encased in plaster-of-Paris; the right foreleg of the animal protruded through the dressing. This leg was carefully shaved, and again made aseptic with bichloride of mercury, and finally with ether. The dog was now ready for the operation. With the assistance of Dr. James E. Kelley, Visiting Surgeon to Charity Hospital, and also Drs. Charles D. Roy, C. Stephenson and J. D. Wood, members of the house staff, we proceeded to the operation upon the patient, which was performed before the matriculates of the Post-Graduate School and Hospital. There were also present Dr. Huntington, of Sacramento, Cal., and Dr. J. F. Winn, of Richmond, Va., and the hospital staff. No one else was present.

Two elliptical incisions were made down to the fracture, four inches in length, removing the old cicatrix and cicatricial tissue about the ununited ends of the bone, together with an elliptical piece of the soft parts. With a saw the ends of the bones were freshened, leaving a space about one inch between them. The portions removed proved to be eburnated, and more like ivory than bone.

Fig. 7.

My asssistants, Drs. Plimptom and Mooney, now prepared the limb of the patient for the next step in the operation, by enveloping it in a plaster-of-Paris bandage, commencing six inches above the incision, and extending to the upper third of the thigh. The foot and ankle were also covered with a plaster-of-Paris bandage.

While they were skillfully preparing this part of the dressing, we were preparing the dog. An incision was made through the skin, as represented in Fig. 7, for the purpose of cutting a piece which would accurately fit the elliptical shaped wound in the patient's leg. The elbow was now quickly excised; the radius and ulna were severed one-half inch in front of the elbow-joint, and the humerus three inches above it, and removed, see Fig. 8, *B*, leaving all soft parts.

Fig. 8.

The extremity, near the paw, was amputated, leaving a piece of bone one inch in length, Fig. 8, *A*, attached to a branch of the brachial artery among the soft parts.

The attachment of the biceps tendon was detached from the bone and loose superfluous muscular tissue removed. In the dog the nutrient artery enters the bone one inch in front of the elbow-joint. Cutting the bone, as indicated above, saved the nutrient artery from injury, and secured the nutrition to the fragment of bone, from which we had hoped that new bone would be thrown out, and at the same time stimulate the human bone to a reparative effort.

The dog was placed by the side of the patient's leg, the head toward the patient.

An aluminum dowel-pin was passed through the medullary

Fig. 10.

cavity in the long axis of the bone. This I think was a mistake. A steel pin inserted into the solid portion of the bone would not interfere with the circulation so much. See Fig. 10. The piece of bone *A*, Fig. 8, was placed between the ends of the bone *B*, *B*, of the patient, as seen in Fig. 11. The bones were crowded together, the dowel-pin entering the bones of the patient above and below. A silver wire was passed around the entire graft, (See Fig. 11,) and securely tied. This held the bone securely in

Fig. 11.

place. Fig. 11 shows the artery giving off its nutrient branch to the grafted bone, *A*. Muscle was stitched to muscle, and skin to skin, the parts being evenly coaptated. *C* is the humerus of the dog.

Fig. 12, is a diagram showing the bone, *A*, in place between

Fig. 12.

the bones of the boy, *B*, *C*. It also shows the dowel-pin, wire, main artery, and nutrient artery. *D*, represents the dog's leg stitched to the soft parts of the patient, *E*. Band iron was bent and adjusted over the wound, from the upper plaster cast to the lower one of the boy's foot, thus leaving room for dressing. A large drainage tube was inserted for drainage, which opened posteriorly. A few turns of the plaster-of-Paris bandage secured the iron rods to the leg. The wound was dressed antiseptically.

Through the entire operation the most rigid antiseptic methods were carried out. Constant irrigation prevented the possibility of wound infection. Having in Drs. Plimpkin and Mooney two most efficient dressers, and working two teams, one for dressing and one for operating, the hands of the operator did not need to come in contact with the plaster-of-Paris or septic dressings. The operation can be performed in one hour with efficient dressers.

The operation appears difficult and complicated, but is quite simple when understood. Many mistakes at the first operation were corrected in this, and many which occurred in this could be corrected in another. The operation of open incision for club foot, which I first performed in 1879 and published in 1880, was perfected only after twenty operations had been done. And so it is with all first operations involving complicating mechanics.

The patients (for we must now say patients) were put to bed. Both recovered from the anæsthetic rapidly. Small doses of morphine were used for both, from time to time, to allay, not so much the pain, as the uneasiness caused by the forced confinement. After three days this uneasiness passed away, and from that time on the dog and patient became friends, administering to each other's comfort—the patient by feeding and playing with the dog, and the dog by vigorous wags of the tail which showed her appreciation of the kindness.

I neglected to say that before the operation was performed the vocal cords of the dog had been carefully severed, under ether, to prevent any disturbance of the patient. At the end of two weeks, however, the cords had again reunited, and the voice of the dog sounded fully as strong as before the operation. The only pain caused to either patient was the twitching of the muscles of the dog as she shrank in her bed from the loss of adipose tissue. This might have been prevented by a simple procedure at the time of operation. But this was a new development which had not occurred in the first case, and which we were not fully prepared to meet when first discovered in this.

On the sixth day the case was dressed in the presence of Drs. Newman, Stewart, Wooley, and Professor Prince Morrow, of the University Medical College, and Visiting Surgeon to Charity Hospital.

The wound was found perfectly healed by primary union, without a single drop of pus. Only for the difference in the color of the skin it would have been difficult to detect the line of union. On the eighth day we again dressed and the union was still perfect and more firm. Finally at the end of eleven days, there was an apparent shrinkage of the dog in the dressings. This allowed of motion, and it became evident that the graft would be pulled from its attachment within a few days. Consequently, much as I desired to continue the experiment, I concluded as a prospective act of humanity to sever the bond of union. I was prepared to do this the moment that I discovered that any surgical interference would become necessary, which would inflict additional pain to either, in order to continue the experiment.

The dog was chloroformed during the operation.

While the graft was being trimmed, and the leg of the patient dressed, Dr. Kelley skillfully secured the artery and nicely stitched up the stump of the dog's leg. She was then placed in bed and cared for by the nurse. As the graft was trimmed down to the parts still attached a free oozing of blood took place, through the graft, which demonstrated the fact that union had taken place and that circulation had been established between the patient and the dog. Both patients rapidly convalesced. The boy spent his time writing letters to his friends and reading the papers and postal cards from persons praying that the effort to save his leg might be a failure.

The wound was dressed and the graft examined daily. At the end of five weeks it was discovered that the bone showed no further sign of uniting, and desiring to give the boy every chance for union of the fracture it was removed. The rods, also, were removed, and the ends of the patient's bones placed firmly together, hoping to secure union because of the stimulation produced by the graft. The bone graft was irregularly covered with a new growth of bone, thus proving, I believe, that an effort had been made to unite the fracture.

This was the result of eleven days' contact, whereas at least thirty days are required for bony union to take place.

The canal of the bone was also filled with a new growth of bone, excepting where the dowel-pin passed through.

The average temperature recorded in the patient was about $99\frac{1}{2}^\circ$, in the deg $99\frac{4}{5}^\circ$. The average pulse of the boy was about 95. The normal temperature of the dog is above a hundred, that of the human being $98\frac{1}{2}^\circ$ F. The temperature of the dog fell to below a hundred and that of the boy rose to near a hundred, or the same as that of the dog, where it remained for weeks. The pulse of the boy rose and the dog's fell until they beat nearly the same number of beats per minute, varying from ninety to one hundred and ten. The boy ate, slept, and felt well. There was no sepsis. Whether this peculiar condition of temperature and pulse was due to the interchanging of blood between the animal and the patient, I am unable to say; further observation is necessary to verify it.

After the eleventh day, owing to the plaster-of-Paris accidentally getting into the wound, pus for the first time was seen. This rapidly disappeared.

The operation had a two-fold object: first, to establish the fact that large masses of soft parts could be transplanted from an animal to man; second, to unite an ununited fracture by a section of bone from the dog. We have succeeded in demonstrating the first proposition, but we have partially failed in the other in so far as the actual growing of the bone into place is concerned. This was due entirely to a defect in the dressings. The principle of transplantation established means much to humanity; its application will be found useful in many cases which now defy the best efforts of the most skillful surgeons in the world.

Among the cases suitable for the application of the principle are those cases of fractures which resist all efforts for their union, and which must necessarily result in amputation; ulcers of a particular class which can be cured by no means known to surgery.

Scalps ripped from the heads of factory girls by machinery. Months and often years have been taken to skin-graft back the scalp to cover the skull, and numerous friends have been flayed to supply the material.

Thiersch's method of skin-grafting has been a step in advance of the older methods, but a martyr must be found to submit to the flaying. A dog would be found better adapted for the work as hair could be transplanted with the flap. Sloughing, following amputations in the upper third of the tibia resulting in cicatrical contraction with indolent granulations covering the end of the stump caused by the bad circulation from pressure are now cured. But how? By amputation at the knee-joint or else so near to it that an artificial limb cannot be worn without a useful knee-joint. Animal transplantation should be resorted to before amputation is performed.

If circulation could be established between opposite species, the elements of whose blood differed, without injury to either, a step would be taken which might lead to the relief of many a sufferer. Then large masses of tissue could be grafted from an animal to man, the circulation of the animal furnishing that which the patient could not supply, as in bone-transplantation. Or in grafting of soft parts the circulation of the dog would keep alive the graft until it had become firmly united to the patient, then it could be severed.

In bone-transplantation it was expected that in four or five weeks the animal would have thrown out a provisional callus and at the same time stimulate the fracture to repair. Figures 13 and 14 seem to substantiate that theory. A dog was selected because the elements of its blood very closely resemble that of man. The reparative energy of a dog is very strong and his power of endurance great. No unnecessary cruelty is inflicted and aside from the confinement but little suffering occurs.

Of course it is uesless to reply to those who have denounced the operation as cruel and unnecessary. Those who understand the motive which actuates the surgeon, can comprehend how, with all sympathy for the brute, his sacrifice of limb maybe demanded for the good of his master, man. They, too, can appreciate the reluctance of the surgeon to inflict wanton suffering, whether upon man or brute, and can understand how such an operation only seemed commendable when a more than commensurate benefit was promised. To those whose eyes are blind to human suffering, and whose sympathies are all for the brute, I have nothing to say.

It will perhaps be remembered that this poor lad demanded that every means should be exhausted which promised relief before amputation should be resorted to. He still demands it, and the demand is one which a humane surgeon should consider before resorting to an operation which would involve the loss of a limb and possibly life.

## Figures and Tables

**Fig. 5. f1:**
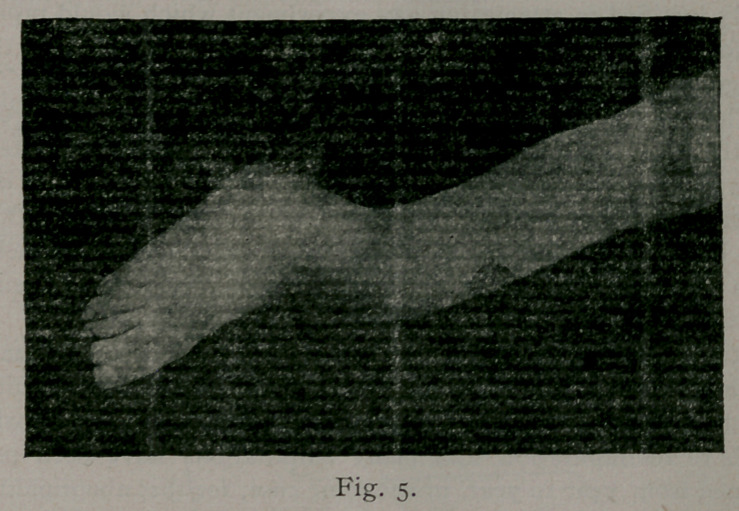


**Fig. 7. f2:**
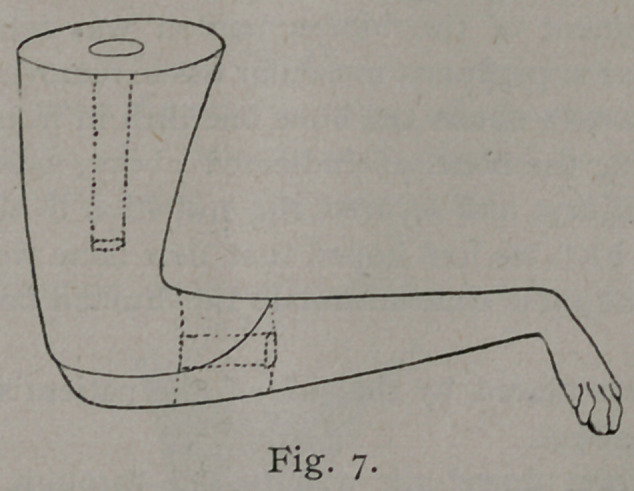


**Fig. 8. f3:**
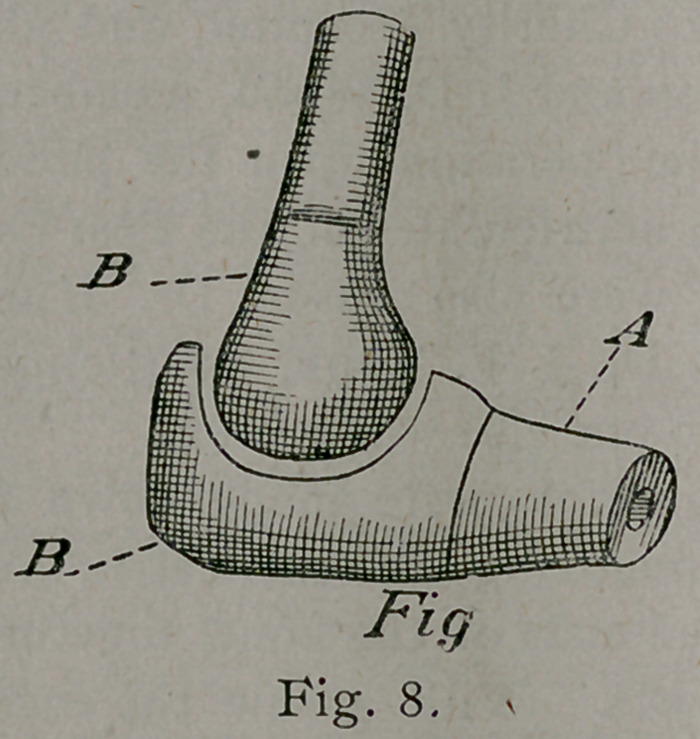


**Fig. 10. f4:**
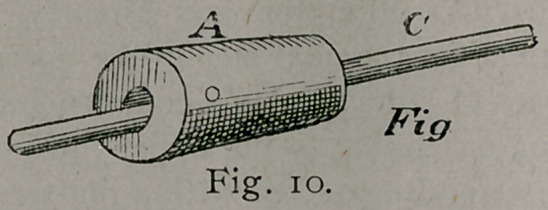


**Fig. 11. f5:**
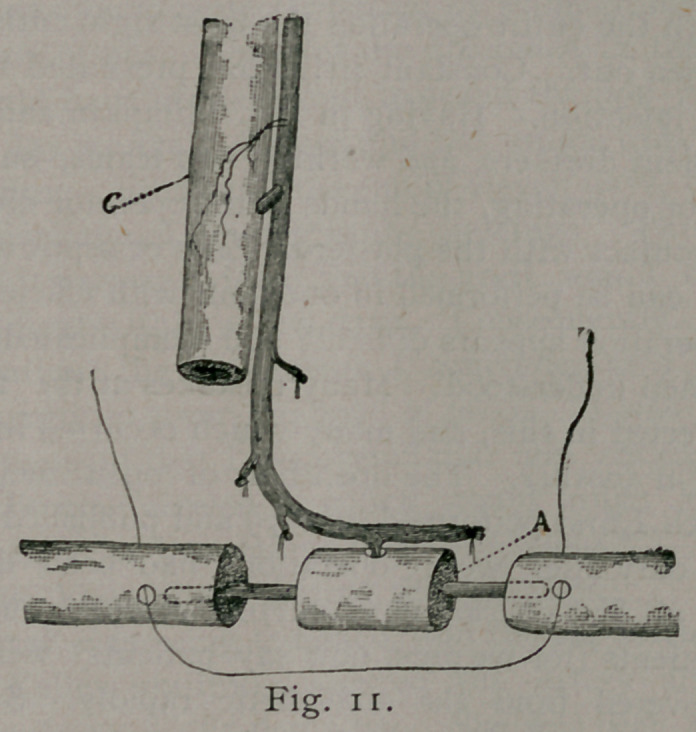


**Fig. 12. f6:**